# Acute exacerbation of COPD: Physiotherapy practice and factors that influence management

**DOI:** 10.4102/sajp.v80i1.2106

**Published:** 2024-12-23

**Authors:** Motheo Phalatse-Taban, Heleen van Aswegen

**Affiliations:** 1 Department of Physiotherapy, Faculty of Health Sciences, University of the Witwatersrand, Johannesburg, South Africa

**Keywords:** COPD exacerbation, physiotherapy, assessment, breathing exercises, exercise therapy, communication, advocacy, psychological support

## Abstract

**Background:**

Acute exacerbation of chronic obstructive pulmonary disease (AECOPD) is a leading cause of morbidity and mortality in South Africa. Physiotherapy practice and factors that influence management of patients with AECOPD are unknown.

**Objectives:**

To explore physiotherapy practice in the management of patients with AECOPD in South African private healthcare settings and to identify and describe factors that influence physiotherapy patient management.

**Method:**

The study adopted a qualitative descriptive design using semi-structured interviews. Purposive and snowball sampling was used to identify physiotherapists working in private healthcare in three South African provinces. Individual interviews were conducted face-to-face or via telephone and transcribed verbatim. Content analysis was done using an inductive approach.

**Results:**

Participants (*n* = 9) working in private hospitals reported that their management is based on patient-specific needs assessment. Treatment interventions included various respiratory physiotherapy techniques and exercise rehabilitation strategies. Patient education on self-management of disease symptoms featured prominently. Enablers of physiotherapy management included supportive workplace relations, conducive work environment, physiotherapists’ competence, familial support and patient cooperation. Barriers identified included limited communication, nurses’ attitudes, work environment, disease burden, mental health challenges and limited professional development opportunities.

**Conclusion:**

Physiotherapists provide individual needs-based care to patients with AECOPD. Various enablers and barriers to physiotherapy patient management have been identified.

**Clinical implications:**

Advocacy for physiotherapy, better communication between multidisciplinary team members and recognition of the need for psychological support are important factors to address to enhance the care provided to patients with AECOPD.

## Introduction

The number of cases of chronic obstructive pulmonary disease (COPD) for patients of all ages increased by 117% from 1990 to 2019 in sub-Saharan Africa mainly because of household air pollution from solid fuels and tobacco use (Alemayohu et al. 2023). In Africa, tuberculosis (TB) and human immunodeficiency virus (HIV) are known risk factors for the development of COPD (Abdool-Gaffar et al. 2019). Chronic obstructive pulmonary disease is characterised by airflow obstruction, hyperinflation and poor pulmonary gas exchange (Troosters et al. 2023). Various infectious and environmental factors lead to acute exacerbation of COPD (AECOPD) (Ko et al. 2016). Loss of lung function and increased mortality are associated with AECOPD (Abdool-Gaffar et al. 2019). Management of patients with AECOPD includes the use of bronchodilators, corticosteroids, antibiotics, oxygen therapy, non-invasive ventilation and physiotherapy (Abdool-Gaffar et al. 2019; Ko et al. 2016).

The South African Thoracic Society advocates pulmonary rehabilitation as an effective management strategy for those with COPD (Abdool-Gaffar et al. 2019). Although pulmonary rehabilitation does not improve lung function, lung mechanics or gas exchange, it targets the systemic effects of COPD such as sarcopenia and deconditioning (Abdool-Gaffar et al. 2019). Pulmonary rehabilitation is a multidisciplinary intervention that incorporates structured exercise training, education, dietary management and psychosocial support (Abdool-Gaffar et al. 2019). The routine use of standalone manual airway clearance techniques, although safe during AECOPD, is not recommended by the South African Thoracic Society (Abdool-Gaffar et al. 2019). These techniques may be beneficial to selected patients who present with excessive secretions and ineffective cough (Abdool-Gaffar et al. 2019). In such cases, airway clearance using positive expiratory pressure (PEP) therapy is recommended (Abdool-Gaffar et al. 2019).

No evidence is available regarding the physiotherapy management of patients admitted to hospital with AECOPD in South Africa. In the South African private healthcare setting, patients admitted with AECOPD are usually referred by the treating physician to a physiotherapy practice for treatment and rehabilitation. In many cases, the referring physician will have specific requirements with regards to the patients’ physiotherapy treatment. The current physiotherapy management of patients with AECOPD is unknown. In addition, the factors that influence physiotherapists’ ability to perform evidence-based management on patients admitted with AECOPD to private healthcare facilities are unknown.

The aim of this qualitative study was to describe current physiotherapy practice and explore the factors that influence South African physiotherapists’ management of patients with AECOPD admitted to private hospital settings.

## Research methods and design

### Study design

A qualitative, descriptive study design was used in which face-to-face or telephonic interviews with participants were conducted, using a semi-structured interview guide.

### Setting

The participants were physiotherapists who worked in three provinces in South Africa, namely Gauteng, Mpumalanga and North West. These provinces were selected as the researchers were aware of physiotherapists who worked in private practice in these provinces and could be invited to participate in the interviews.

### Study population and sampling

Physiotherapists who manage patients with AECOPD on a weekly or monthly basis in a private hospital setting and who had a minimum of 3 years’ work experience were invited to participate in the study. Initially, purposive sampling was used to identify potential participants through personal contacts of the researchers. Thereafter, snowball sampling was used to identify further potential participants from participants who had completed the interviews.

### Data collection

Participant recruitment started in April 2020 and concluded in November 2020. Interviews were initially conducted face-to-face between one of the authors (M.P.-T.) and each participant. Because of enforced travel restrictions around the coronavirus disease 2019 (COVID-19) pandemic, subsequent interviews were conducted telephonically. An information sheet and consent form were sent via email to the physiotherapists who indicated an interest in participating in the study. After written consent for participation and audio-recording was obtained, a date and time for each interview was arranged. On the day, a study-specific questionnaire was completed by each participant shortly before commencement of the interview. Characteristics (e.g. clinical experience and experience working with patients with AECOPD) and demographic data (e.g. age and gender) of participants were captured using this questionnaire. A semi-structured interview guide, comprising three broad questions, was used to guide the interview. Based on participant responses, additional follow-up questions were posed to elicit further information as needed.

Interviews were recorded using a traditional audio recorder. In the case of telephonic interviews, the phone was operated on the ‘loudspeaker’ function with the audio recorder in proximity to accurately record the telephonic interview. The researcher noted her own reactions and experiences during the interviews to prevent bias or contamination of the research findings by her own personal experiences. The ontological assumptions made for this study were the reality that adults with AECOPD are hospitalised at least annually, and that each patient presents with their own signs and symptoms which impacts physiotherapy management of individual patients. Clinical practice guidelines in the setting where the physiotherapist works might also influence their patient management approach. Interviews were conducted until the same comments arose in individual interviews, at which point, no more interviews were conducted.

### Data analysis

Inductive content analysis was done as not much has been published about physiotherapy management of patients with AECOPD in South Africa (Vaismoradi, Turunen & Ondas 2013; Vears & Gillam 2022). The preparation phase involved verbatim transcription of recordings following each interview by one of the authors (M.T.-P.). The transcripts were read and re-read for immersion in the data to obtain a sense of the whole. The data were organised through iterative coding and re-coding to identify meaning units and codes, comparing and grouping codes into content categories and identifying sub-categories. Manifest content was analysed. The number of similar codes in each sub-category is reported. Independent coding was done by both authors who met afterwards to discuss the codes and content categories that were identified from the data. Analysis was completed through synthesising and connecting the categories to create a narrative report. Supportive quotes from participants are included in the narrative report (Vaismoradi et al. 2013; Vears & Gillam 2022).

Rigour was ensured by selecting a few participants (one from each province) for member checking. The selected participants were sent their own recorded interview and transcript to listen to and read through. This was done for verification of the accuracy with which their viewpoints were captured. Dependability was ensured through evaluation of the process of deriving meaning units to categories with an experienced researcher. Confirmability was achieved through triangulation and transferability through description of the characteristics of the participants (Vaismoradi et al. 2013).

Descriptive statistics were used to present quantitative data. Data were summarised as means and standard deviations and minimum and maximum ranges. Analysis was done using the Statistical Package for Social Sciences (SPSS) version 27.0 (IBM^®^ Corporation, Armonk, New York, United States).

### Ethical considerations

Ethical clearance was obtained from the University of the Witwatersrand Human Research (Medical) Ethics Committee (certificate number: M190865). Permission was obtained from the respective physiotherapy practice owners in Gauteng, Northwest and Mpumalanga provinces to interview their staff. Participant and patient anonymity was ensured by removing all personal identifiers from the transcripts and assigning to each transcript a participant number.

## Results

**FIGURE 1:**
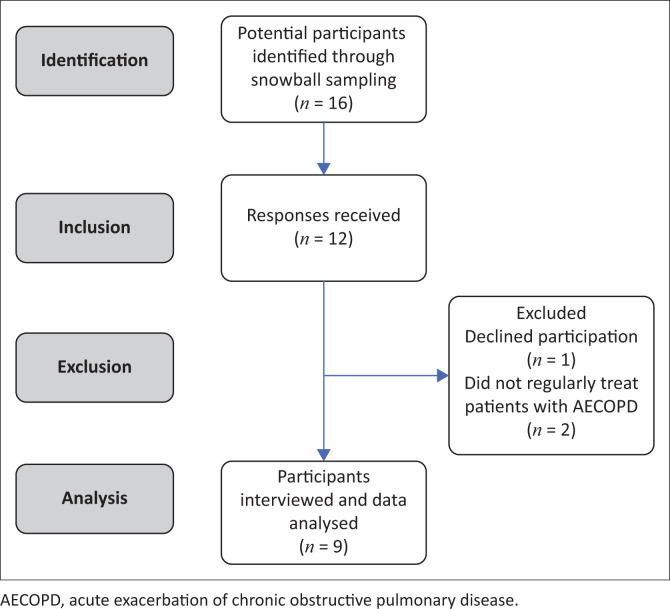
Summary of participant recruitment for the study.

**FIGURE 2:**
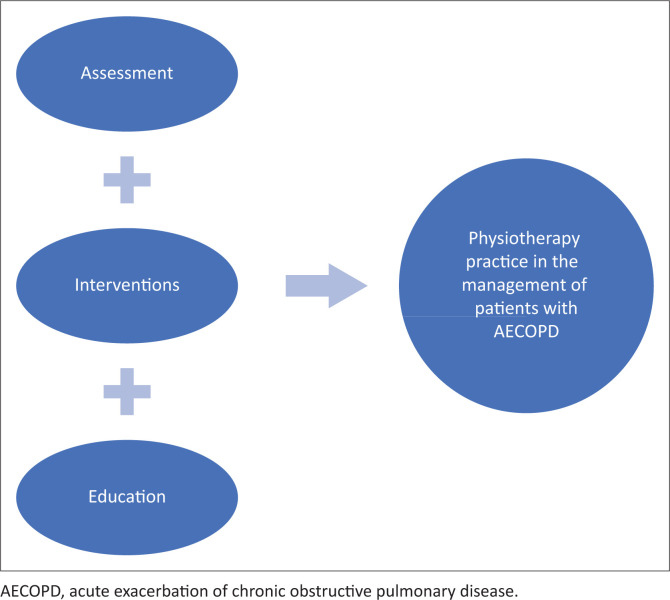
Illustration of components that form part of physiotherapy practice in the management of patients with acute exacerbation of chronic obstructive pulmonary disease in private healthcare settings.

**FIGURE 3:**
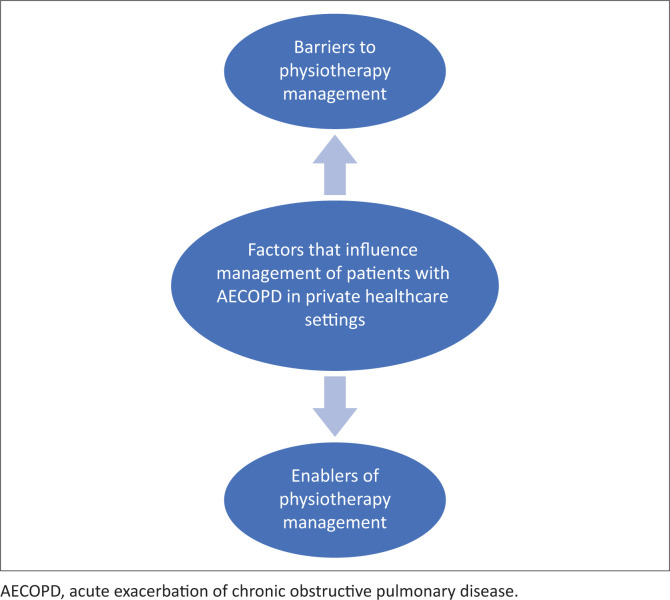
Illustration of factors that influence physiotherapy management of patients with acute exacerbation of chronic obstructive pulmonary disease in private healthcare settings.

Nine individual interviews were conducted over the 8-month period. Figure 1 summarises participant recruitment for the study. The participants had a mean of 7.3 (2.5) years general work experience (range: 3–10 years) and 5.2 (2.1) years of work experience specifically with patients with AECOPD (range: 3–9 years). Three sub-categories were identified related to the content category of physiotherapy practice and two sub-categories related to factors that influence physiotherapists’ management of patients with AECOPD in private healthcare settings (Figure 2 and Figure 3).

### Physiotherapy practice

Participants reported that they performed individual needs-based assessment of their patients with AECOPD which informed the interventions that they selected for patient management. Patient education on self-management of disease symptoms featured prominently. Table 1 presents the categories, codes and supportive participant responses related to physiotherapy practice.

**TABLE 1:** Sub-categories, codes and supportive participant responses related to physiotherapy practice in the management of patients with acute exacerbation of chronic obstructive pulmonary disease in private healthcare settings.

Sub-categories	Codes	Supportive quotations
Assessment	Needs-based (*n* = 7)	‘I would not just do percussions and vibrations and nebulise, I’ll assess first. If the patient is weak, you do strengthening, according to the assessment’. (Participant 2, 34 years, female) ‘We start off by doing assessment first and then make sure that they [are] at the right oxygen level’. (Participant 8, 29 years, female) ‘I’ll do my physical assessment; I’ll do auscultation and check on the monitors as to how the patient has been responding [*to treatment*]’. (Participant 9, 29 years, male)
Interventions	Management of respiratory symptoms (*n* = 8)	‘The vibromat actually helps to relax the muscles … it does give the patient a degree of relaxation which I think helps their breathing …’ (Participant 1, 29 years, female) ‘… with severe shortness of breath I do a lot more ACBT and autogenic drainage. Sometimes if they come in severely short of breath at rest, I don’t even do a full cycle, I just focus more on breathing control, pursed lip breathing, longer expiration …’ (Participant 3, 33 years, female) ‘… stay away from percussions because sometimes percussions make them worse, so do just vibrations and breathing techniques’. (Participant 8, 29 years, female) ‘… with the normal chest physio [*therapy*] manual modalities or techniques, if they can tolerate that, in combination with nebulisation of course to bring about that little bit of relief’. (Participant 9, 29 years, male)
	Mobilisation and exercise therapy (*n* = 8)	‘I think mobilisation plays a huge part in how a patient recovers’. (Participant 1, 29 years, female) ‘… mobilisation is definitely also important. If the patient is weak, you do strengthening …’ (Participant 2, 34 years, female) ‘… trying to optimise their thoracic movement, whatever thoracic movement they have available’. (Participant 3, 33 years, female) ‘… get them out of the bed and see how much they can do, with oxygen’. (Participant 5, 31 years, female) ‘We brought [*gym*] cycles into the hospital for in case we have a COPD patient … that has been the most rewarding thing’. (Participant 8, 29 years, female)
Education	Pathophysiology of disease (*n* = 1)	‘I will do some education with them. I find that most of them don’t understand really the nature of the disease and that it’s progressive … I try to explain a bit of the physiology, then it seems like they are more compliant with exercises’. (Participant 6, 31 years, female)
	Lifestyle behaviour and disease management (*n* = 3)	‘You still have to educate them about smoking cessation, and thinking about the environment that we are working at [mining], there’s a lot that can affect them at this moment’. (Participant 8, 29 years, female) ‘Checking if they’re using their inhalers correctly …’ (Participant 4, 32 years, female) ‘… trying to teach them the coping mechanisms, because most of them tend to get too anxious of the activity before they even do it’. (Participant 5, 31 years, female)

COPD, chronic obstructive pulmonary disease.

### Enablers of physiotherapy management

Participants identified supportive workplace relations, conducive work environment, comprehensive healthcare funding, physiotherapists’ competence, familial support and patient cooperation as enablers to their management of patients with AECOPD in private healthcare settings. Table 2 summarises these six sub-categories and their codes and supportive participant responses related to enablers of physiotherapy management.

**TABLE 2:** Sub-categories, codes and supportive participant responses related to enablers of the physiotherapy management of patients with acute exacerbation of chronic obstructive pulmonary disease.

Sub-categories	Codes	Supportive quotations
Workplace relationships	Healthcare workers attitudes (*n* = 8)	‘We work with doctors who are pro-physio[*therapy*], who see a need for physio[*therapy*]. It gives you some room to say: “You know I learnt this new thing, can I try it with a patient?”’. (Participant 3, 33 years, female) ‘In our medical ward, the nurses try to even get the patient to sit at the edge of the bed …’ (Participant 8, 29 years, female)
	Communication (*n* = 3)	‘We can get a lot of information from them [*doctors*] on [*the patient’s*] prognosis [*and*] how far can we push’. (Participant 6, 31 years, female) ‘If you have a good relationship with [*the*] doctor, I think it’s much easier to actually get feedback from the doctor …’ (Participant 1, 29 years, female)
Conducive environment	Equipment (*n* = 6)	‘… that you do have the necessary equipment that you need to see the patient, to treat the patient effectively’. (Participant 7, 28 years, male) ‘… get a little step so that they climb up and they climb down …normally it would be like a colourful thing around their bed and the cycling and Theraband …’ (Participant 8, 29 years, female)
Healthcare funding	Outpatient follow-up (*n* = 4)	‘A lot of our patients have medical aid and access to funds to look after their health. We have the ability to get our patients [*to*] see you as an outpatient if it is required, whereas it might be difficult if they didn’t have those funds available’. (Participant 3, 33 years, female)
Physiotherapist’s competence	Knowledge and skills (*n* = 4)	‘The fact that I expanded my knowledge of COPD patients, my experience, my handling skills make it easier, my clinical reasoning make it easier …’ (Participant 4, 32 years, female) ‘Definitely just knowledge overall because I know what I need to do for this kind of patient’. (Participant 5, 31 years, female)
Familial support	Encouragement (*n* = 3)	‘Family members, if they are present, it’s definitely an enabler, to help encourage the patient, calm the patient’. (Participant 4, 32 years, female)
Patient cooperation	Knowledge of condition (*n* = 3)	‘The patient’s own knowledge of their condition. They have to have an idea, an understanding and it makes the [physiotherapy] treatment much easier’. (Participant 5, 31 years, female)

COPD, chronic obstructive pulmonary disease.

### Barriers to physiotherapy management

Physiotherapy management of patients with AECOPD was influenced negatively by factors such as limited communication from healthcare workers and patients, nursing staff attitudes, work environment, disease burden, mental health challenges and limited continuous professional development opportunities. These sub-categories, codes and supportive quotations from participants are presented in Table 3.

**TABLE 3:** Sub-categories, codes and supportive participant responses related to barriers to physiotherapy management of patients with acute exacerbation of chronic obstructive pulmonary disease.

Sub-categories	Codes	Supportive quotations
Communication	Healthcare workers (*n* = 3)	‘There’s no real communication [*with the doctor*] and you don’t feel like you can approach him and ask him questions about the patient’. (Participant 6, 31 years, female)
	Patients (*n* = 1)	‘The little bit of Zulu that I do use helps, but language is also sometimes a huge barrier’. (Participant 4, 32 years, female)
Attitudes	Nursing staff (*n* = 8)	‘Probably the biggest one is the nursing staff, where most of my frustration comes from. I would, for example, feel like a patient would do well in the chair and the nurse will [*say*] “but he’s very weak, he can’t” and I’ll say, “but if we do it together, we can do it”. Nurse responds “No, my back is sore today”’. (Participant 1, 29 years, female) ‘I mostly have challenges with getting nursing staff to help mobilise patients because they think that it’s our job’. (Participant 4, 32 years, female)
Work environment	Rivalry between physiotherapy practices (*n* = 1)	‘Fighting over patients … the doctor refers the patient to you but last year they were seen by the other practice’. (Participant 4, 32 years, female)
	Workload (*n* = 3)	‘You have a long list that limits the time that you spend with the patients … it makes sense because it’s an acute [*care*] hospital, not a rehabilitation facility’. (Participant 8, 29 years, female)
Disease burden	Severity of exacerbation (*n* = 3)	‘If the condition is very severe, they struggle to do almost anything’. (Participant 6, 31 years, female) ‘… a patient with acute exacerbation, very short of breath, panicked, very anxious, their ability to participate in treatment is very difficult in the beginning’. (Participant 3, 33 years, female)
Mental health challenges	Lack of support (*n* = 3)	‘In patients with chronic COPD … you find a lot of burden because they have nobody to offload with, or to support [them] in certain aspects of their daily living’. (Participant 5, 31 years, female)
	Anxiety (*n* = 1)	‘So with acute exacerbations I find patients’ anxiety is a big barrier’. (Participant 3, 33 years, female)
	Physiotherapist’s mood (*n* = 1)	‘Personal issues can also have an effect, because if I’ve got issues at home I’m not going to be [*in*] a very good mood to motivate a patient’. (Participant 2, 34 years, female)
Continuous professional development	Limited opportunities through professional society (*n* = 3)	‘I would like to see a lot more cardiopulmonary courses from our society … even if it’s just online courses based on what we’re dealing with’. (Participant 5, 31 years, female)

COPD, chronic obstructive pulmonary disease.

## Discussion

This qualitative study contributes to the existing knowledge gap about South African physiotherapy management of hospitalised patients with AECOPD, specifically in the context of private healthcare. Physiotherapy patient management is initiated through individual needs-based assessment. Individualised plans of care are created for patients with AECOPD based on the findings of the assessment. Intervention strategies used in patient care are aimed at assisting with clearance of retained secretions, relieving of shortness of breath, addressing the systemic effects of COPD, and increasing patients’ knowledge and understanding and self-management of their condition. This concept of holistic patient care resonates with the guideline on standards of physical therapy practice published by World Physiotherapy (World Confederation of Physical Therapy 2011).

Some participants shared that as part of their assessment, they reviewed their patient’s oxygen therapy to verify whether it corresponds to the recorded prescription parameters in their files. Although oxygen therapy is a life-saving intervention, it can have detrimental effects on the outcomes of patients with AECOPD if not correctly administered. Oxygen therapy is, at times, incorrectly administered by nurses working in medical wards (Hickey 2007) and several errors in oxygen therapy procedures are reported, particularly the absence of monitoring of oxygen therapy delivered to patients (Neves & Lobão 2012). This underscores the importance of including assessment of oxygen therapy parameters delivered to patients with AECOPD into physiotherapy management.

Respiratory physiotherapy techniques such as various breathing exercises, manual chest clearance techniques (e.g. percussions and vibrations) and nebulisation with bronchodilator therapy were used by most participants. The use of chest wall vibration (applied manually or with a mechanical vibrator) to aid secretion clearance in patients with copious secretions is supported in the systematic review by Hill, Patman and Brooks (2010). Although evidence to support the routine use of airway clearance techniques in patients with AECOPD is inconsistent, Holland (2014) highlights that many studies do not account for the different phenotypes of COPD. This implies that some patients with AECOPD may experience clinical benefit from such management strategies while others may not. It is therefore important that physiotherapists assess each patient’s response to treatment and adapt their management approach accordingly. The individualised plans of care created by participants for patient management suggest that most do consider their patients’ responses to treatment received.

All participants reported that they included exercise therapy and education together with these respiratory therapy interventions in their patient treatment sessions. This practice aligns with the South African Thoracic Society guidelines of not using respiratory techniques as standalone interventions in the management of patients with AECOPD (Abdool-Gaffar et al. 2019). None of the participants mentioned that they used PEP therapy as part of patient management, despite it being recommended in the South African Thoracic Society guidelines (Abdool-Gaffar et al. 2019) and a recent systematic review on physiotherapy management of patients with AECOPD (Torres-Sánchez et al. 2017). Oscillating PEP therapy is delivered through flow-regulated (e.g., Flutter) or pressure-regulated (e.g., bubble PEP) devices that create resistance upon expiration during spontaneous breathing to temporarily splint open unstable airways to facilitate clearance of retained secretions (Fagevik Olsén et al. 2021). An important advantage of oscillating PEP therapy is that patients can carry on with this therapy throughout the day after they receive instruction on how to use the device correctly. South African physiotherapists should thus consider including PEP therapy in their management of patients with AECOPD.

Exacerbation of COPD is associated with worsening hypoxaemia, increased dyspnoea and acute-on-chronic systemic inflammation. Limb muscle weakness and decreased functional exercise tolerance develop because of decreased activity, deconditioning, nutritional deficits and use of higher doses of systemic corticosteroids (Kim et al. 2019; Troosters et al. 2023). Low intensity exercise training improves movement efficiency and desensitises the sensation of dyspnoea (Troosters et al. 2023). Bronchodilator administration prior to exercise training and exercising while on oxygen therapy are strategies that optimise the ventilatory ability of patients with AECOPD and enable them to participate in strength training activities (Troosters et al. 2023). Exercise therapy is therefore an important management component of patients hospitalised for AECOPD (DeGaris & Osadnik 2020; Kim et al. 2019; Troosters et al. 2017, 2023). The inclusion of exercise therapy, at times while the patient is on oxygen, as routine management for patients with AECOPD by participants in this study, suggests the translation of evidence into their clinical physiotherapy practice. A concern is that only one participant incorporated chest wall mobilisation techniques in patient care. Lung hyperinflation is worsened during episodes of acute exacerbation and leads to the chest wall being maintained in a hyperinflated state. This causes a decrease in the already impaired chest wall mobility of patients with COPD that impacts on the efficiency of their respiration and ventilation. Chest wall mobilisation techniques, added to patient education and walking exercise, result in improved respiratory muscle strength, thoracic excursion and thoracic range of movement in patient with severe COPD (Tsui et al. 2023) and should be considered in the management of patients with AECOPD. Factors that contribute to participants not including chest wall mobilisation as part of evidence-based physiotherapy patient management need further exploration. The limited access to professional development opportunities related to AECOPD that some participants alluded to may be a contributing factor.

Multidisciplinary team involvement in the care of patients with COPD is advocated in the South African Thoracic Society guidelines (Abdool-Gaffar et al. 2019). Some participants shared that enablers to their patient management included working in an environment where doctors and nurses were supportive of physiotherapy involvement in patient care, and they collectively communicated openly regarding their patient management plans. Conversely, some participants experienced negative attitudes especially from nursing staff towards their involvement in patient care and found communication with patients’ physicians prescriptive and unwelcoming. These findings suggest that multidisciplinary team collaboration in patient care is not always optimal in private healthcare settings.

Participants who work in an environment where sharing of knowledge and skills was welcomed, felt more competent to manage patients admitted with AECOPD. Others felt limited in their professional development as they struggle to access relevant articles in scientific journals and feel that not enough continuous professional development (CPD) courses are made available through their professional society. All participants were employees in private physiotherapy practices. It is suggested that they explore the possibility of journal subscription with their employers to overcome this barrier in professional development. The availability of accredited physiotherapy courses focussed on the management of patients with AECOPD hosted by South African professional societies and academic physiotherapy departments needs further exploration.

Some participants felt that the high patient workload in the settings that they work and rivalry between private practices that work in the same hospital setting, posed barriers to quality of patient care delivered. One participant acknowledged that their mood, especially if they experience stressors in their personal lives, influenced the quality of care provided to their patients. The impact of the abovementioned factors on quality of physiotherapy patient care in private healthcare settings deserves further exploration.

Participants mentioned that their patients’ mental health and level of familial support received could be an enabler or barrier to physiotherapy management. Patients who understood their condition were seen to be more appreciative of the contribution that physiotherapy made to their recovery from AECOPD. The presence of family members with patients during physiotherapy sessions was enabling of a better quality of treatment as patients seemed to be less anxious and more willing to actively participate. This resonates with the notion that positive social support is associated with improved disease management behaviours in COPD (Lenferink, Van der Palen & Effing 2018). Conversely, stresses from living with COPD such as reduced mobility and fear and embarrassment of symptoms, can disrupt relationships, increase isolation and place a burden on relationships and healthcare workers (Lenferink et al. 2018). These stresses were observed by participants in some of the patients that they manage during AECOPD and impacts negatively on patient cooperation and physiotherapists’ mental health. One participant shared that language was a barrier to physiotherapy service delivery if a patient was not able to understand the language spoken by the physiotherapist. South Africa is a multi-lingual country with 11 official languages, and therefore it is inevitable that this barrier may be encountered by healthcare workers from time to time. Approaching other healthcare staff or visiting family members or friends to act as interpreters during physiotherapy sessions may assist in fostering a better physiotherapist–patient relationship.

### Strengths and limitations

A strength of qualitative descriptive research is that insight is gained about the subjective experiences of participants, a more in-depth understanding of the context that shapes their experiences is developed, and power is given to their voices in reporting the study findings (Ho & Limpaecher 2023). A limitation is that one of the authors works in a similar setting to the participants (private hospital in South Africa) and had similar previous interactions with treating physicians and other members of the multidisciplinary team and may have inadvertently influenced participants’ responses during the interviews. Every effort was made to avoid this influence on the study findings. The study design and number and location of participants limit the generalisation of findings. The findings lay the foundation for future studies that describe in-depth the scope of physiotherapy practice in the management of patients with AECOPD in private South African healthcare sectors.

## Conclusion

The findings suggest that physiotherapists working in private healthcare settings provide individual needs-based care to patients with AECOPD. The quality of care provided is enhanced by supportive colleagues, conducive work environments and the physiotherapist’s level of clinical expertise. Familial support provided to patients and patients’ level of understanding of their condition either enable quality of physiotherapy care received or pose limitations to care provided. Better communication between multidisciplinary team members and patients, and recognition of the need for psychological support for patients, when indicated, are important factors to address to enhance the care provided to adults with AECOPD in private healthcare settings.
